# Supplementary figures and images for: Protein Arginylation Is Regulated during SARS-CoV-2 Infection
Source: Viruses. 2023 Jan 19;15(2):290. doi: 10.3390/v15020290 (PMC9964439; doi:10.3390/v15020290)

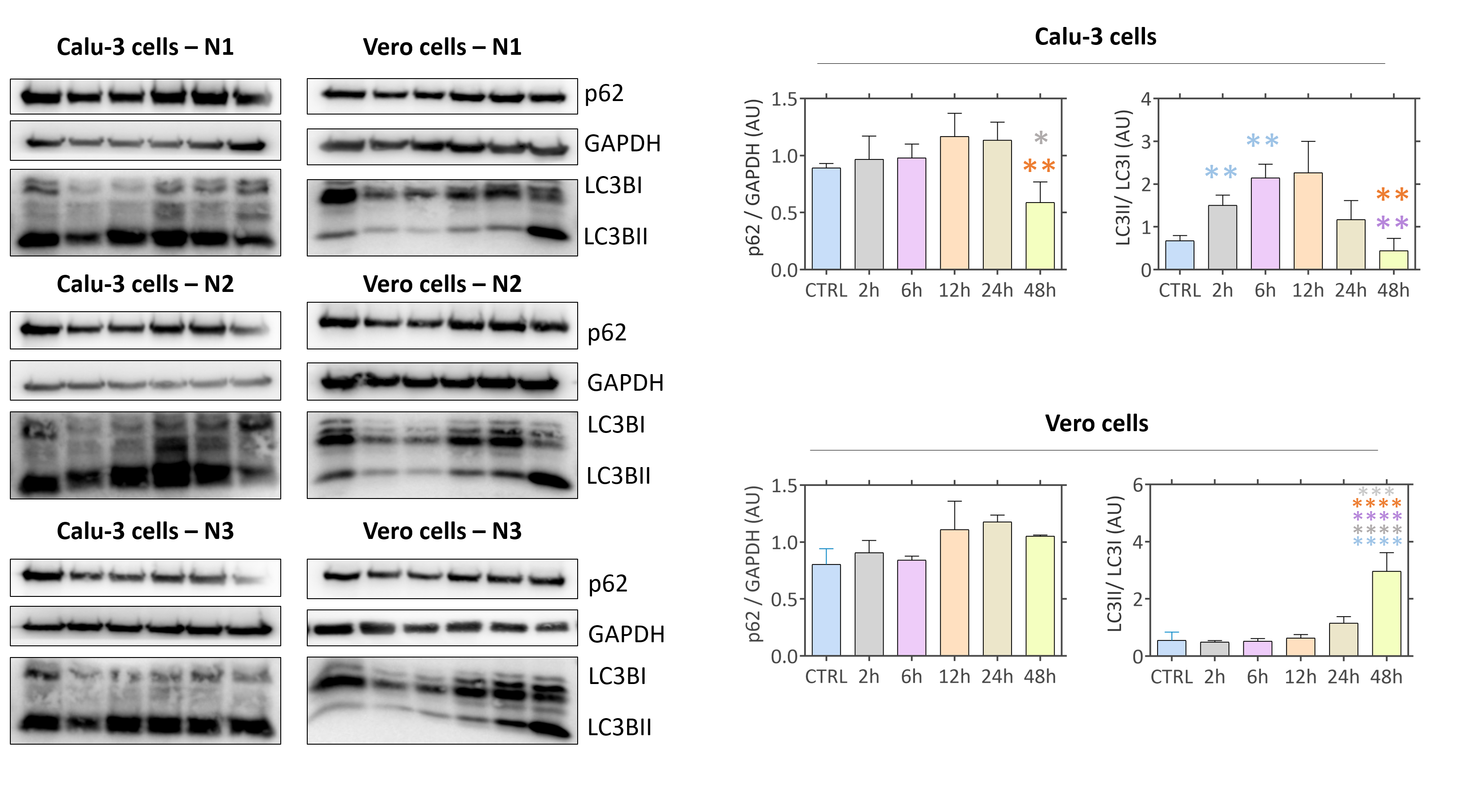

Supplement: Supplementary file 1 [file viruses-15-00290-s001.zip › Figure S1.png]

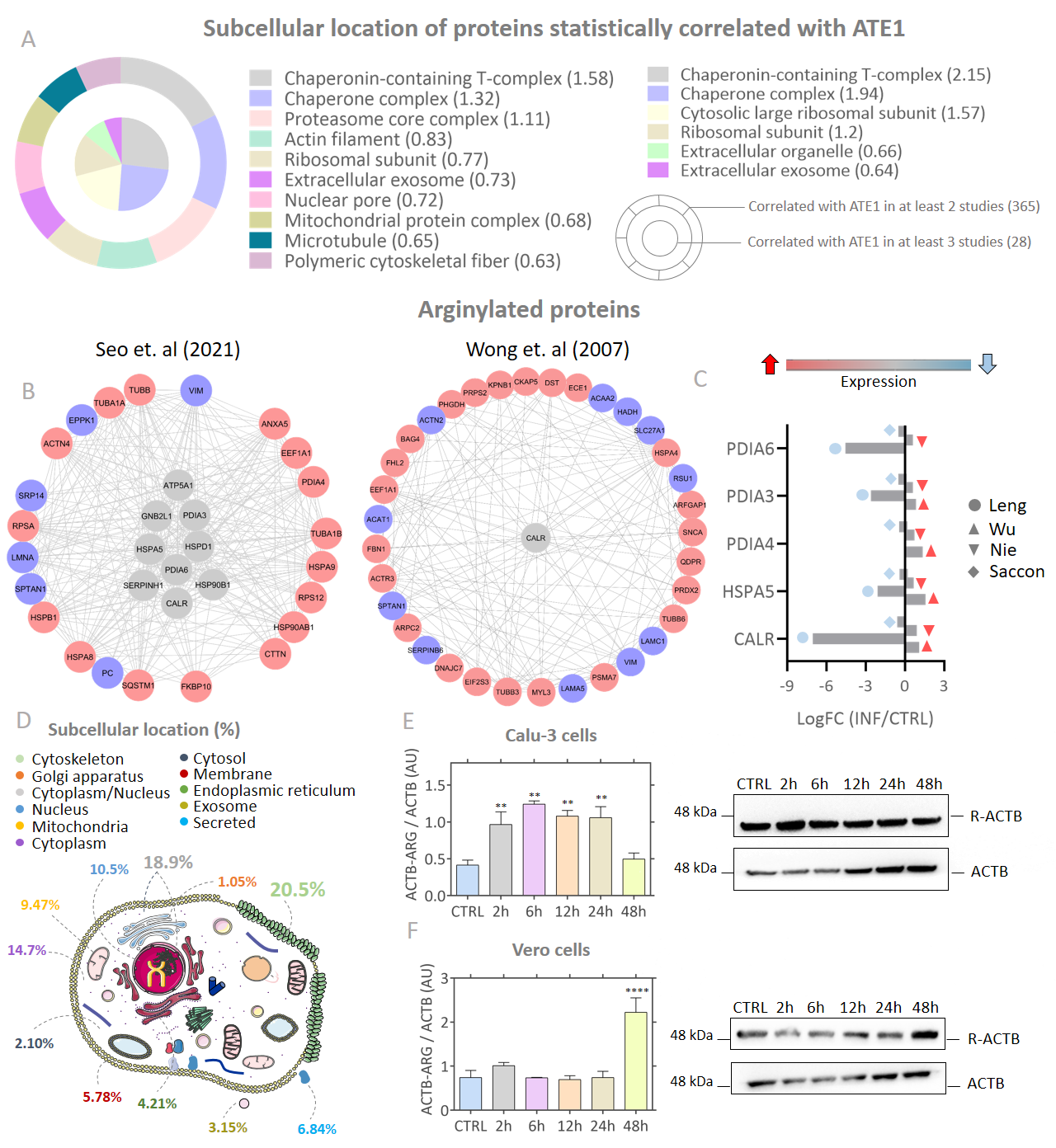

Supplement: Supplementary file 1 [file viruses-15-00290-s001.zip › Figure S2.tif]

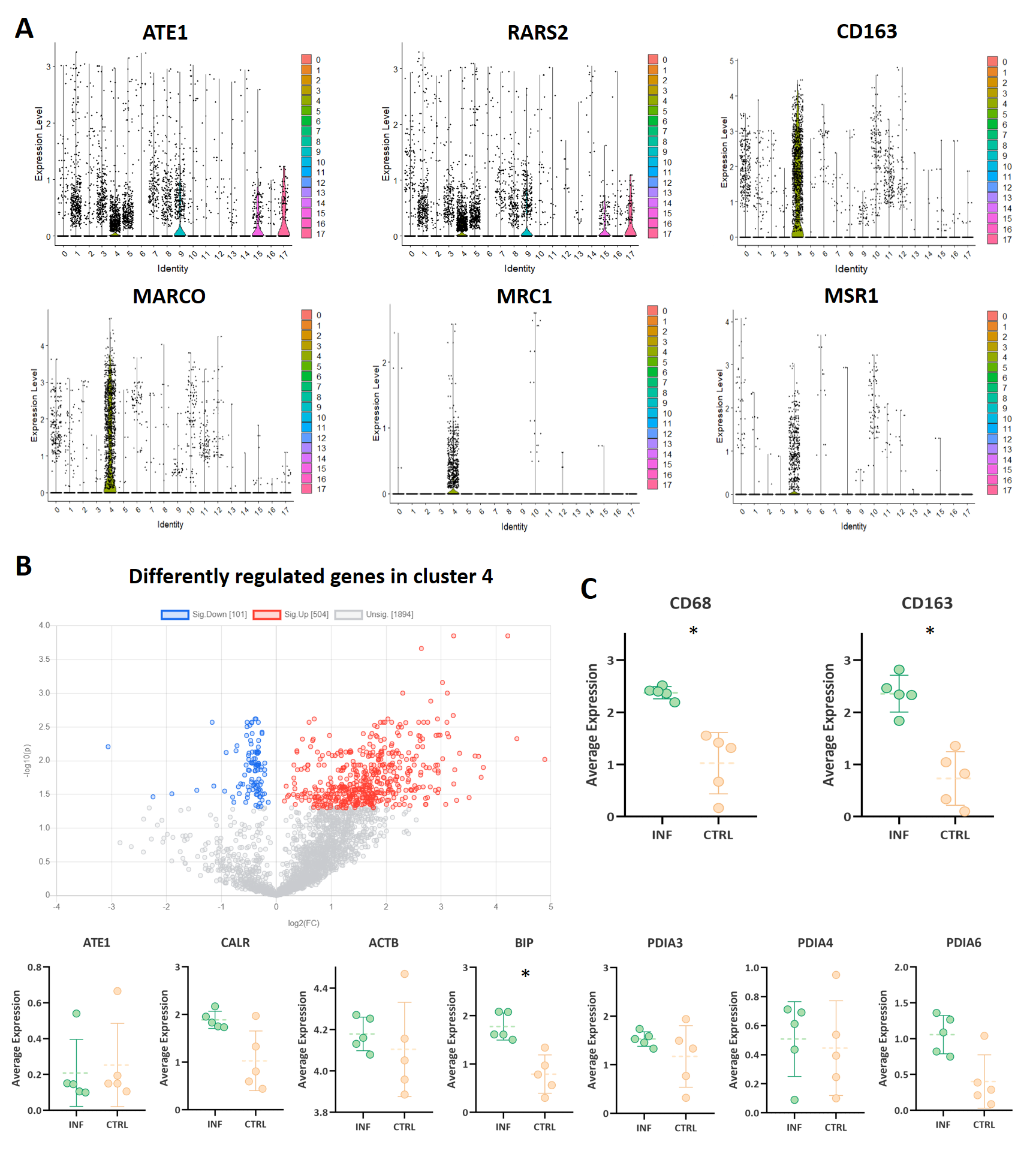

Supplement: Supplementary file 1 [file viruses-15-00290-s001.zip › Figure S3.tif]

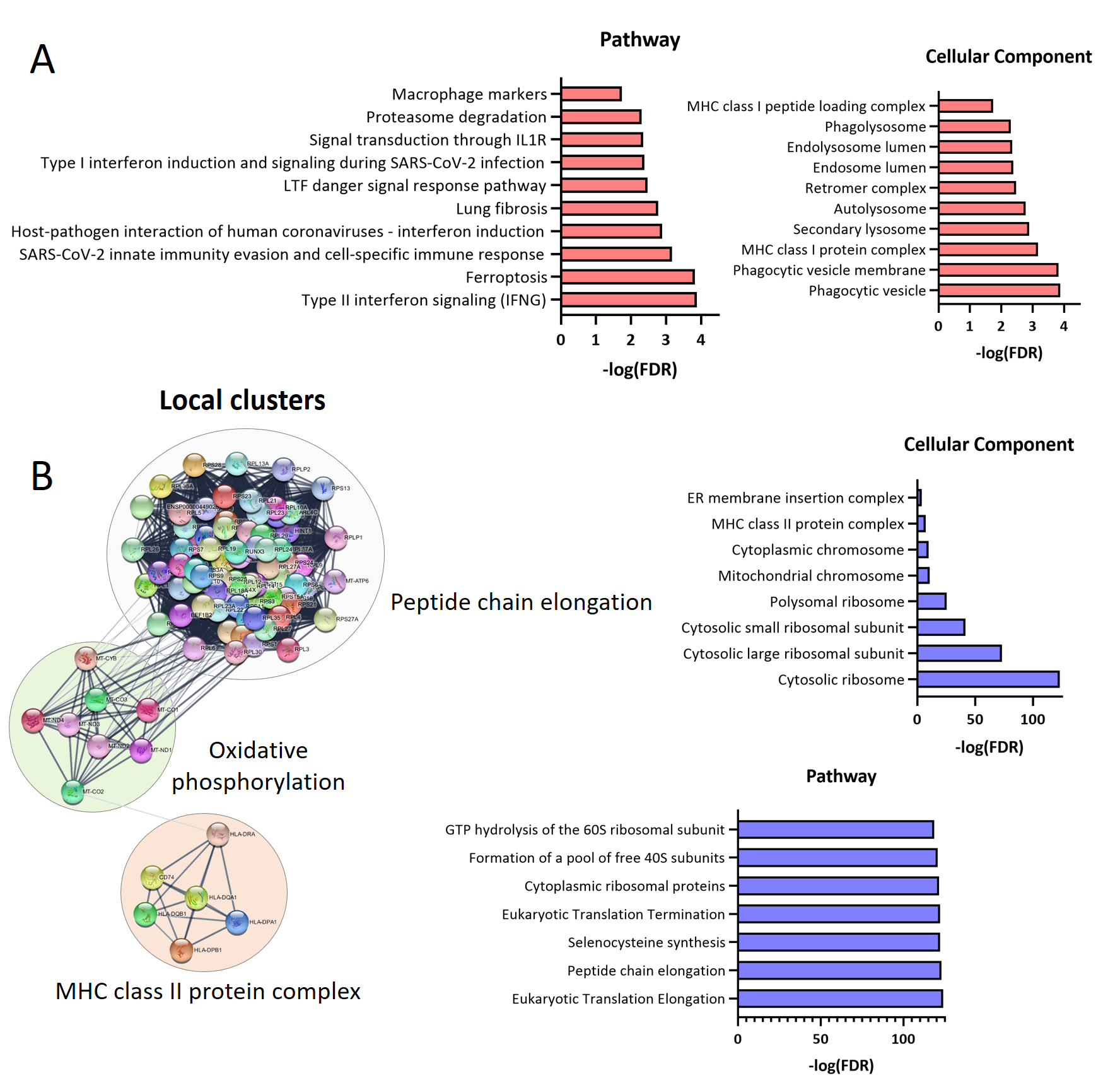

Supplement: Supplementary file 1 [file viruses-15-00290-s001.zip › Figure S4.tif]

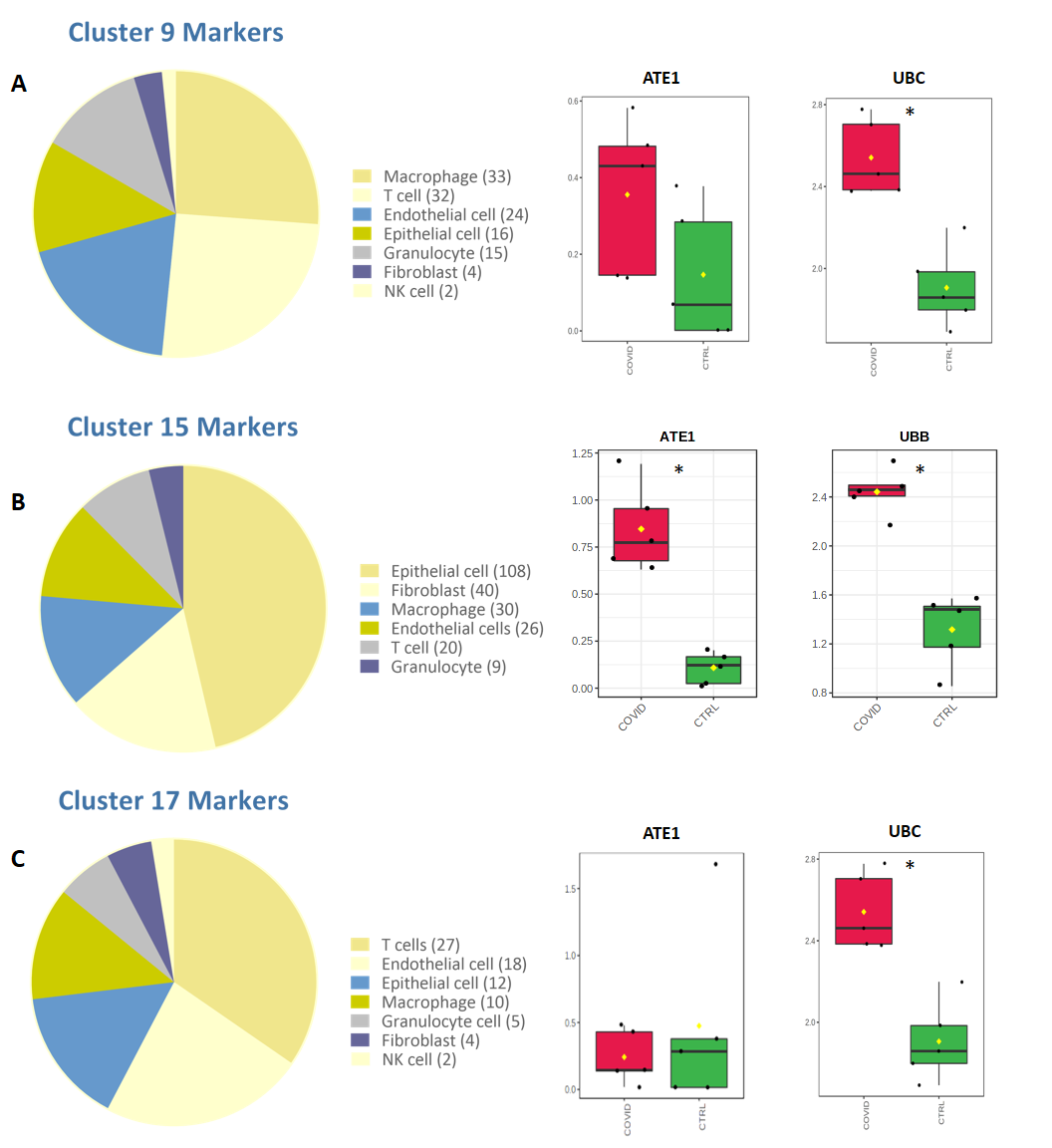

Supplement: Supplementary file 1 [file viruses-15-00290-s001.zip › Figure S5.tif]

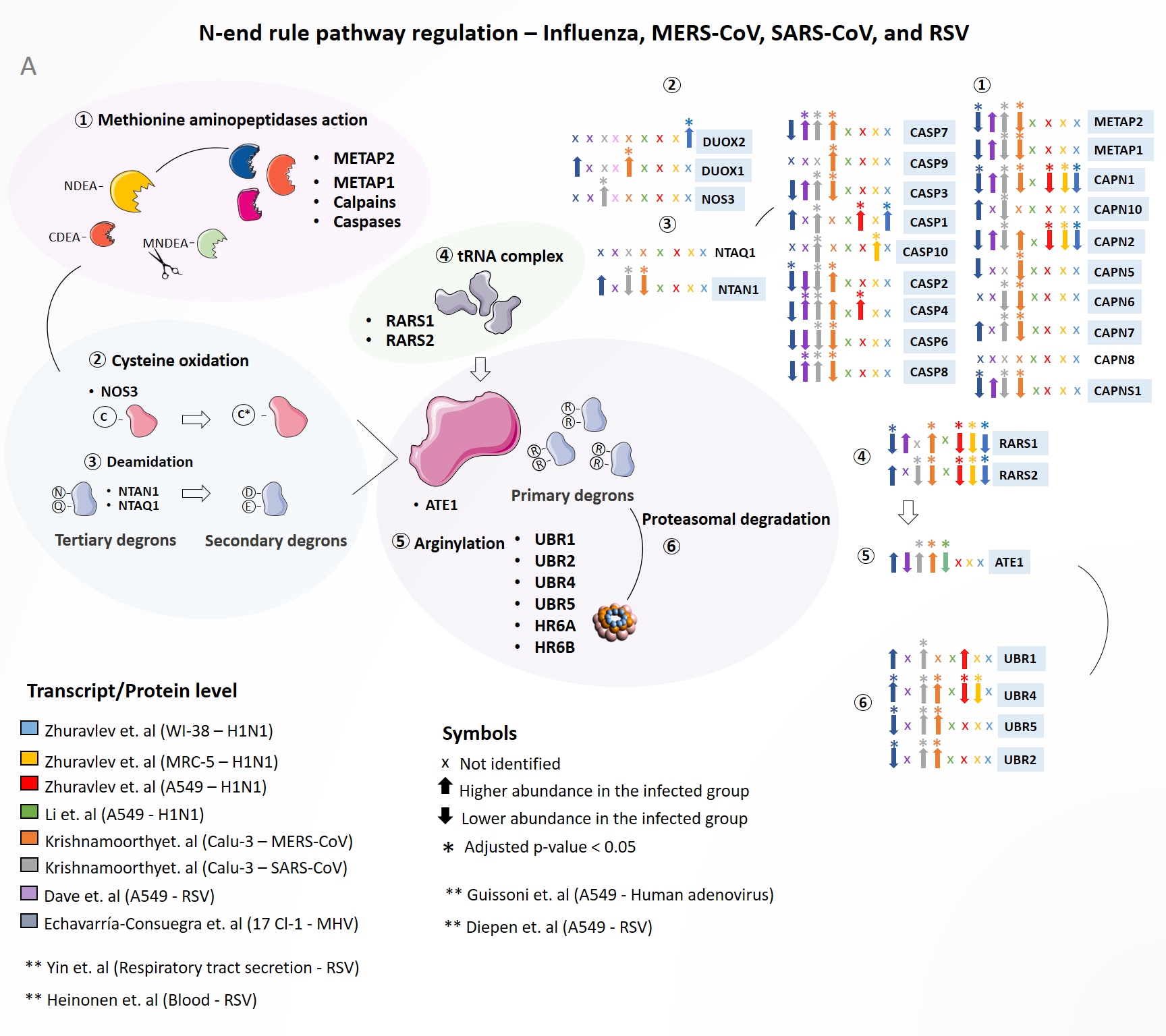

Supplement: Supplementary file 1 [file viruses-15-00290-s001.zip › Figure S6.tif]
